# The optimality of the standard genetic code assessed by an eight-objective evolutionary algorithm

**DOI:** 10.1186/s12862-018-1304-0

**Published:** 2018-12-13

**Authors:** Małgorzata Wnętrzak, Paweł Błażej, Dorota Mackiewicz, Paweł Mackiewicz

**Affiliations:** Department of Genomics, Faculty of Biotechnology, University of Wrocław, ul. Joliot-Curie 14a, 50-383 Wrocław, Poland

**Keywords:** Evolution, Genetic algorithm, Mutation, Optimization, Standard genetic code, Translation

## Abstract

**Background:**

The standard genetic code (SGC) is a unique set of rules which assign amino acids to codons. Similar amino acids tend to have similar codons indicating that the code evolved to minimize the costs of amino acid replacements in proteins, caused by mutations or translational errors. However, if such optimization in fact occurred, many different properties of amino acids must have been taken into account during the code evolution. Therefore, this problem can be reformulated as a multi-objective optimization task, in which the selection constraints are represented by measures based on various amino acid properties.

**Results:**

To study the optimality of the SGC we applied a multi-objective evolutionary algorithm and we used the representatives of eight clusters, which grouped over 500 indices describing various physicochemical properties of amino acids. Thanks to that we avoided an arbitrary choice of amino acid features as optimization criteria. As a consequence, we were able to conduct a more general study on the properties of the SGC than the ones presented so far in other papers on this topic. We considered two models of the genetic code, one preserving the characteristic codon blocks structure of the SGC and the other without this restriction. The results revealed that the SGC could be significantly improved in terms of error minimization, hereby it is not fully optimized. Its structure differs significantly from the structure of the codes optimized to minimize the costs of amino acid replacements. On the other hand, using newly defined quality measures that placed the SGC in the global space of theoretical genetic codes, we showed that the SGC is definitely closer to the codes that minimize the costs of amino acids replacements than those maximizing them.

**Conclusions:**

The standard genetic code represents most likely only partially optimized systems, which emerged under the influence of many different factors. Our findings can be useful to researchers involved in modifying the genetic code of the living organisms and designing artificial ones.

## Background

The standard genetic code (SGC) describes how 64 possible codons encode 20 amino acids and the stop translation signal. This fundamental discovery [1, 2] explained how the genetic information stored in the DNA molecule can be transmitted to the protein world. The specific properties of the code, e.g. that similar amino acids tend to have similar codons assigned [3], inspired many scientists to formulate various hypotheses concerning its origin and evolution [4–7], such as: (i) the stereochemical hypothesis, (ii) the coevolution hypothesis, and (iii) the adaptive hypothesis. These hypotheses indicate different factors as the main forces responsible for the present structure of the SGC, although it is not inconceivable that all these factors played main roles at different stages of the standard genetic code evolution [6].

The stereochemical hypothesis postulates that a high affinity between amino acids and their codons/anti-codons or other nucleotide aptamers and oligomers had a decisive impact on the SGC structure [8–13]. The main argument against this explanation lies in the fact that such interactions have been found only in very few cases. Because of the lack of strong experimental evidence this hypothesis cannot be considered the basic explanation for the structure and evolution of the SGC. The coevolution hypothesis claims that the present structure of the SGC is a reflection of the expansion of prebiotic pathways for the biosynthesis of amino acids [14–21]. According to this scenario, the SGC evolved from its ancestral form, which encoded only a small number of amino acids produced by simple biochemical reactions. In the consecutive evolutionary stages other amino acids were incorporated into the code simultaneously with the evolution of more complex metabolic networks.

The adaptive hypothesis has become quite popular since its formulation [22, 23]. It claims that the structure of the SGC evolved to minimize the effects of amino acid replacements resulting from mutations or translational errors. This concept was investigated by many researchers using many methodologies [24–41]. The main argument for this scenario follows directly from the observed tendency in the SGC of amino acids with similar properties to be located in a close vicinity in the genetic code table and to differ usually by only one substitution in their codons. For example, hydrophobic amino acids are usually encoded by codons with uracil in the second position and hydrophilic amino acids by those with adenine in this position.

To test the adaptive hypothesis, many researchers used various approaches and constructed many models to deal with the extremely huge number of possible theoretical genetic codes. Given 64 codons and 20 amino acids with the stop translation signal, there are 21^64^ ≈ 4 · 10^84^ variations with repetition of 21 elements taken 64 at a time, i.e. 64-tuples of 21-set. Assuming that each code has to encode each out of 21 items, this number is only slightly smaller 1.51 · 10^84^ [42]. If we accept a model of the SGC evolution involving two sets of 32 complementary triplets, where each set coded for 10 amino acids, we will still have a very large number of possible codes: 10^32^ · 10^32^ = 10^64^ [43]. These astronomical numbers of the codes can be reduced only by assuming the evolution of the SGC from the primeval RNY code and the inclusion of specific features: the degeneracy, the wobbling rule, the assignments of aminoacyl-tRNA synthetases to amino acids, the assumption that glycine was the first amino acid, as well as the topological and symmetrical properties [43].

To assess the optimality of the SGC, a comparison with randomly generated theoretical codes was usually performed [24–27, 44]. However, this classic approach seems to be inefficient because even 1 · 10^6^ random codes make only a very small fraction of all possibilities and do not have to be representative for the whole space of theoretical codes. Therefore, genetic and evolutionary algorithms, which try to find the best possible codes under given criteria and compare them with the SGC, seem more suitable for this problem [37, 41, 45–52].

Another important issue in the investigation of the SGC optimality is the choice of amino acid properties, which has to capture the most crucial features for peptides synthetized when the genetic code was emerging. The selection of such properties is not an easy task. Obviously, the optimality cannot be studied in regard to amino acid substitution matrices, commonly used in phylogenetic analyses and sequence alignments, e.g. PAM and BLOSUM matrices, because they include the substitutions that were already selected by the genetic code structure, which makes such analyses tautologous [53]. In agreement with the fact that such types of matrices include not only a component depending on pairwise amino acid similarities but also one resulting from mutability of amino acids [54], which may reflect just the genetic code structure. Physicochemical properties most commonly tested in the SGC optimality are: hydropathy, isoelectric point, molecular volume and polarity [24, 37, 47, 50, 55, 56]. The last one provided the most significant evidence for the error minimization property of the SGC and was used in further analyses by many researchers. However, this and other mentioned properties are only a small sample of all possible characteristics which can be used to describe amino acids. Most probably, many features of amino acids influenced the evolution of the SGC, not just as single factors, but rather as a system of interconnected elements.

Therefore, studying the potential optimality of the SGC as a multi-objective optimization problem and using evolutionary algorithms seems to be a proper approach. Thanks to that, we were able not only to answer the question about the optimization properties of the SGC but also to detect amino acid features that could have had an impact on the SGC structure. Moreover, in order to assess the level of optimality of the SGC in terms of robustness to amino acid replacements, we decided to find not only the codes minimizing but also those maximizing the costs of amino acid replacements, to avoid comparing the SGC with randomly generated codes. We searched for the optimal codes in two groups of codes, one containing the codes with the same codon block structure as the SGC, and the other with codes without such restrictions. As a search method we applied a customized version of the Strength Pareto Evolutionary Algorithm, which is popular and widely used in various optimization problems [57].

We subjected to optimization the costs of all possible changes from one amino acid to another caused by single-point mutations in codons. Such costs can be described by differences in physicochemical and biochemical properties of amino acids. However, there are over 500 amino acid indices quantifying such properties collected in the AAindex database [58], which makes it difficult to choose the most significant, non-redundant and informative ones for given analyses. A good way to overcome these problems is to look for a clustering of these indices in terms of their similarities and to select the most representative index for each cluster. The recent classification of such indices was made by [59] using a consensus fuzzy clustering method. Thus we applied eight amino acid indices representing various amino acid properties obtained by this method to assess the costs of amino acid replacements. Besides the eight-objective optimization, we also carried out single-objective optimizations of all the considered criteria separately, for comparison. The results of this approach showed that it is justifiable to use the multi-objective algorithm because it provides much more information about the features and the structure of the genetic code than the single-objective optimization method.

## Methods

### Models of genetic codes

We considered two models of genetic codes. The first one, the block structure model (BS), preserves the characteristic codon block structure of the standard genetic code and simply permutes the assignments of amino acids between these codon blocks in order to create a new genetic code. The second one, the unrestricted structure model (US), randomly divides 61 sense codons into 20 non-overlapping sets corresponding to 20 standard amino acids, with the assumption that each of these sets is not empty. In both models, the codons assigned to the stop translation signal stay the same as in the SGC for all newly created genetic codes.

### Multi-objective evolutionary algorithms

In order to find the optimized genetic codes, we decided to apply one of the multi-objective evolutionary algorithms (MOEAs). They are widely used in solving optimization problems because of their many advantages, such as simplicity, flexibility and robustness to changing conditions [60]. These algorithms are especially useful in the cases where analytic methods are not able to produce reliable results due to the specific properties of the search space, especially its size.

MOEAs require: (i) a well-defined search space to represent potential solutions, (ii) objective functions to evaluate the quality of solutions, (iii) genetic operators to create new solutions from the set of previously considered ones, and (iv) a selection mechanism to choose solutions for the next generations [60]. The algorithm starts with a population of randomly generated individuals, which are subjected to genetic operators and evaluation. On the basis of the fitness function values, the selection procedure chooses the individuals to constitute the next generation, on which the genetic operators, the evaluation and the selection are applied again. This procedure is repeated until a stopping rule is activated or a stable solution is reached.

The genetic operators are responsible for generating diversity in the population and the selection is supposed to favour better individuals over others for reproduction. Therefore, most of the better solutions from one generation pass to the next one. Certain worse individuals may pass to the next generation as well; it is another way to maintain diversity in the population and to avoid getting stuck in a local optimum.

### Genetic operators

As mentioned before, MOEAs require genetic operators to produce new individuals from the previous generation. Usually two such operators are used: mutation and crossover. Although they are both responsible for producing variation in the population, they differ in meaning and the results of their action. The mutation operator makes small random changes in the individuals to introduce new information into the population. The crossover operator creates new individuals (offspring) by recombining parts of two parents chosen from the previous generation. It is obvious that the form and implementation of these operators depend on the properties of the search space and the way in which potential solutions are presented.

**Fig. 1:**
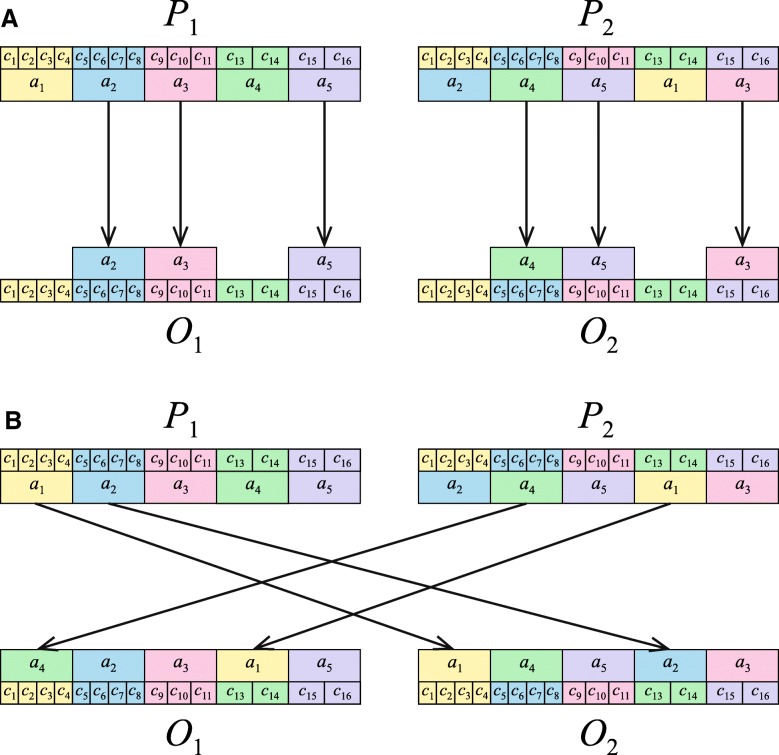
The scheme of the crossover operator for the BS model. **a** Amino acids of the parental code *P*_1_ are selected randomly and assigned to the corresponding codon blocks in the offspring *O*_1_. Similarly, amino acids of the parental code *P*_2_ are also selected randomly and assigned to the corresponding codon blocks in the offspring *O*_2_. **b** The consecutive amino acids of *P*_1_ are selected one by one and assigned to the remaining codon blocks in *O*_2_. Similarly, the consecutive amino acids of *P*_2_ are also selected one by one and assigned to the remaining codon blocks in *O*_1_. When an amino acid is already present in the offspring, the next one in the amino acid set is selected

In our simulations, the genetic codes under the BS model were represented by vectors of 21 elements corresponding to 20 amino acids and the stop translation signal assigned to codon blocks. As the mutation operator, we used a simple swap, which exchanges the amino acids assigned to two randomly selected codon blocks. In the case of the crossover, we adapted the Position Based Crossover (POS) operator [61]. The algorithm of this operator is presented in Fig. 1 and in [51].

**Fig. 2:**
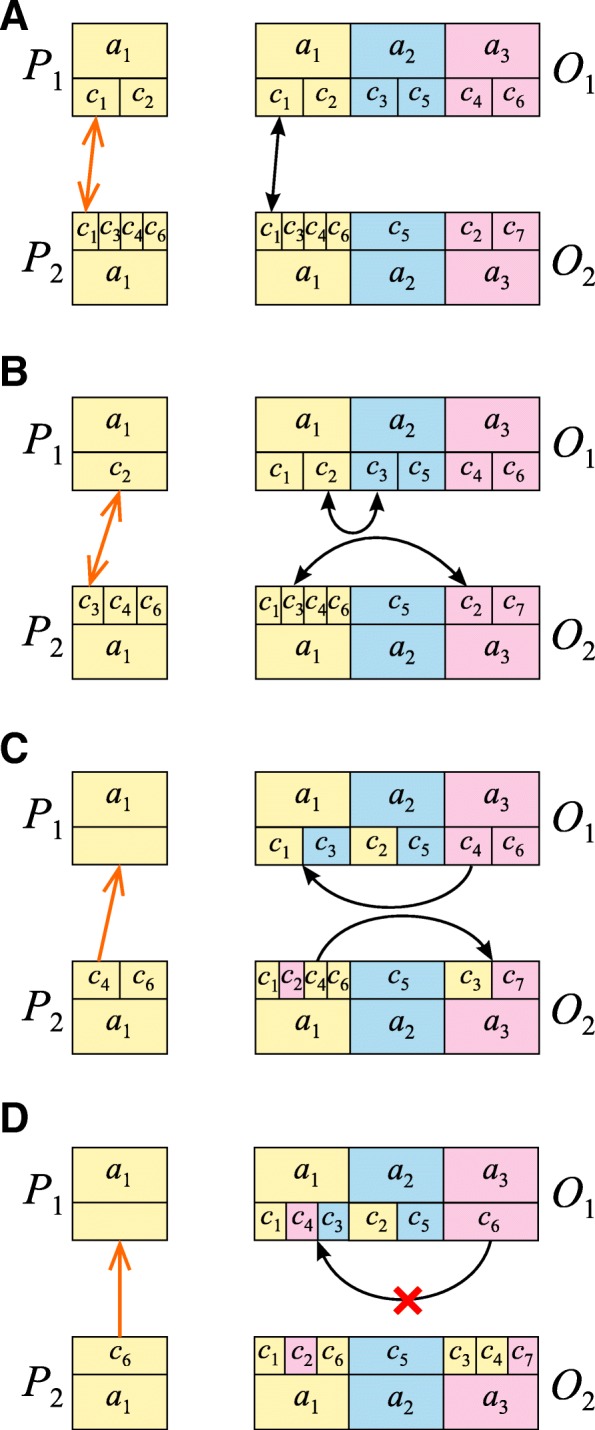
The scheme of the crossover operator for the US model. **a** Two offspring *O*_1_ and *O*_2_, which are identical to their corresponding parents *P*_1_ and *P*_2_, are created. The same amino acid in *P*_1_ and *P*_2_, e.g. *a*_1_ is randomly selected. If this amino acid is encoded by the same codons (the orange arrow), no exchange of these codons is performed between *O*_1_ and *O*_2_ (the black arrow). **b** If this amino acid is encoded by different codons in *P*_1_ and *P*_2_ (the orange arrow), these codons are exchanged mutually within *O*_1_ and *O*_2_ (the black arrows). **c** If there are no codons for the amino acid *a*_1_ in one parent to exchange, e.g. *P*_1_ but the second parent, e.g. *P*_2_ has still a codon for this amino acid (the orange arrow), this codon is moved from other amino acid, e.g. *a*_3_, and assigned to *a*_1_ in one offspring, e.g. *O*_1_ (the black arrow). In the second offspring *O*_2_, this codon is moved from the amino acid *a*_1_ and assigned to the other amino acid *a*_3_ (the black arrow). **d** Codons that are the only ones for the given amino acid in parents (the orange arrow) are not moved in the offspring (the black arrow with the red x)

The genetic codes under the US model were represented by vectors of 64 elements corresponding to codons with assigned respective amino acids. The mutation operator was realized by assigning a randomly selected amino acid to a randomly selected codon, different from the previously assigned one. To guarantee that all amino acids are always represented in the individuals, this procedure was not applied when the selected codon was the only one for a certain amino acid. We additionally used a swap operator which selects at random two codons encoding two amino acids and changes the meaning of all the codons attributed to one of these amino acids for the other amino acid and vice versa. In the case of the US model, we had to propose a different crossover operator from the one used in the BS model; otherwise the offspring might not have inherited their parents’ structures and some amino acids might have even been left out of the offspring’s structure. The full description of this new operator is presented in Fig. 2 and can be found in our paper [51].

### Objectives

As the objectives in the genetic code optimization we considered the costs of all possible changes from one amino acid to another caused by single-point mutations in codons. In choosing the costs, we used the results obtained by [59]. The authors applied a consensus fuzzy clustering method to split more than 500 amino acid indices of the AAindex database [58] into 8, 24 or 40 subsets. They also determined the representatives for each cluster. Therefore, we can assume that the selected parameters are representative for the most relevant amino acid properties. Regarding the computational complexity of our optimization algorithm, we chose eight indices from the AAindex database, which are the representatives of clustering into eight subsets. These indices are: BLAM930101, BIOV880101, MAXF760101, TSAJ990101, NAKH920108, CEDJ970104, LIFS790101 and MIYS990104. They represent diverse amino acid properties, such as: electric properties (isoelectric point and polarity), hydrophobicity, alpha and turn propensities, physicochemical properties, residue propensity (molecular weight, average accessible surface area and mutability), composition, beta propensity and intrinsic propensities (hydration potential, refractivity, optical activity and flexibility). On the basis of the chosen indices we created matrices of squared differences between the values of the given index. To make the comparisons between different objectives easier, these matrices were standardized by dividing each element of the given matrix by the maximum element of this matrix.

### Fitness and objective functions

An important part of each MOEA is to measure the quality of potential solutions in order to guide the search for the most suitable solution to a given problem [60]. This task is realized by a fitness function *F*, which assigns a fitness value to every individual in the population, so that promising solutions could be selected for the next generation.

In a multi-objective optimization problem, the fitness function is based on the values of many objective functions and every individual is described by a vector of values calculated for the respective objective. In our case, to obtain the values for each of the eight objectives, we considered all the possible changes between amino acids resulting from single-point mutations in the codons of the genetic code, similarly to [24, 26]. The squared differences in the given amino acid property between the amino acids before and after mutation were summed up and assigned as the objective function value for the studied objective:

$$ {F}_i(code)=\sum \limits_{<{c}_{1,}{c}_2>\in C}{\left[{p}_i\left({c}_1\right)-{p}_i\left({c}_2\right)\right]}^2, $$

where: *F*_*i*_(*code*) is the value of the objective function for a given genetic code (*code*) and objective *i*, *C* is the set of all pairs of codons which differ only by a single-point mutation, *c*_1_ and *c*_2_ are codons, *p*_*i*_(*c*_1_) and *p*_*i*_(*c*_2_) are the values of amino acid index *i* for the amino acids encoded by the codons *c*_1_ and *c*_2_, respectively.

We compared the vectors of objective values for given individuals according to the well-known Pareto dominance concept [62]. It states that the solution *S*_1_ dominates the solution *S*_2_ if no component of *S*_1_ is worse than the corresponding component of *S*_2_ and at least one component of *S*_1_ is better than the respective one of *S*_2_. However, these conditions are not always fulfilled and then we get a set of equivalent optimal solutions, which are not dominated by each other. The set of optimal solutions, which are non-dominated, i.e. the Pareto set, is denoted as *P*, and its image *F*(*P*) is called the Pareto front [62]. In the SPEA2 algorithm, the fitness value assigned to each individual depends on the number of individuals dominated by the given individual and the number of individuals dominating the given individual [57].

We cannot rule out that the used fitness function is too simple to correctly model the important factors that influenced the evolution of the SGC in terms of the adaptive hypothesis. However, this function is very effective computationally and includes the most relevant amino acid properties that are claimed by this hypothesis. A similar function was applied by other researches, who formulated the adaptive hypothesis. In this work we applied the most advanced fitness function on the basis of such properties. Therefore, we think that this function is appropriate for the studied problem.

### Selection

A new (mating) population is created by selecting individuals for reproduction using the selection operator. We used a tournament selection with the following algorithm:

1. Randomly choose two individuals of the current archive set.
2. Compare the fitness values of the chosen individuals.
3. Copy the individual with better fitness to the mating pool with a probability proportional to its fitness value.
4. Repeat steps 1–3 as many times as the desired number of individuals in the mating pool.

Such algorithm allows for putting not only the most fitted individuals but also some worse ones into the mating pool, which helps in preserving the diversity of the population and avoiding local optima. Tournament selection with a tournament of size 2 and fitness proportional selection by themselves are unlikely to provide sufficient selection pressure for efficient optimisation [63–65]. However, sufficient selective pressure (i.e., elitism) is achieved by the algorithm using an archive where the best non-dominated individuals are kept.

### Strength Pareto evolutionary algorithm

**Fig. 3:**
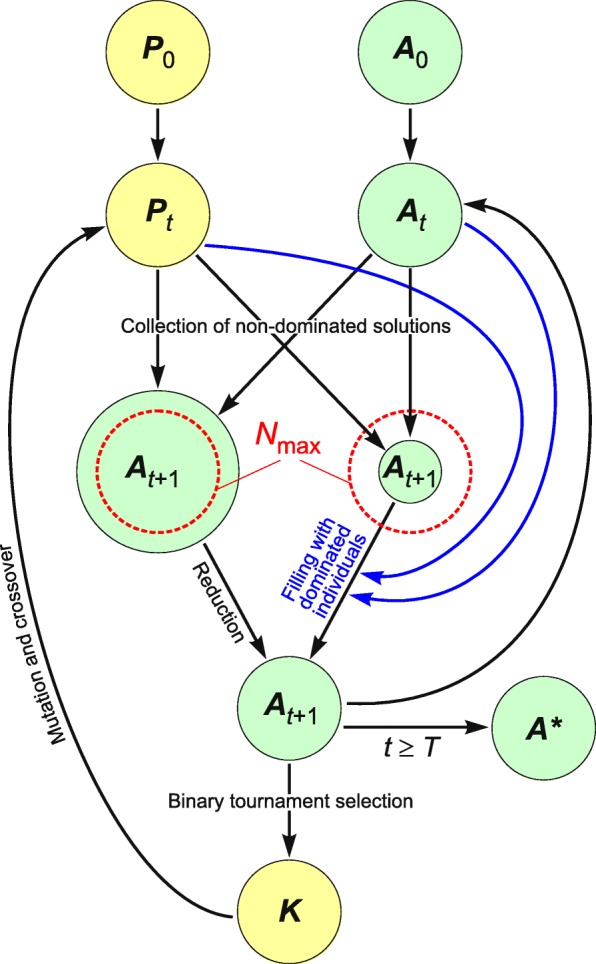
The scheme of the Strength Pareto Evolutionary Algorithm (SPEA2). *N*_max_ is the maximum size of the archive set, *t* - the current number of generations, *T* - the maximum number of generations, *P* - the population of individuals, *A* - the archive set, A* - the final non-dominated set and *K* - the mating pool

To search the space of potential genetic codes, we applied a customized version of the Strength Pareto Evolutionary Algorithm (SPEA2) [57], which is crafted for multi-objective optimization and finds an approximation of the set of optimal solutions to a given problem. The main loop of the SPEA2 algorithm according to [57] is as follows (see also Fig. 3 for a graphical representation):

1. Let *M* be the size of population, *N* - the size of the archive set, *N*_max_ - the maximum size of the archive set, *t* - the current number of generations (steps), *T* - the maximum number of generations, *P* - the population of individuals, *A* - the archive set, A* - the final non-dominated set, *K* - the mating pool, *p*_*m*_ – the probability of applying the mutation operator, *p*_*c*_ – the probability of applying the crossover operator.
2. Set *t* = 0, generate an initial population *P*_0_ with size *M* and an empty archive set *A*_0_ = ∅.
3. Go to the next generation *t:=t* + 1.
4. Calculate values of the fitness function for individuals of *P*_*t*_ and *A*_*t*_ to select non-dominated individuals.
5. Copy all non-dominated individuals of *P*_*t*_ and *A*_*t*_ to *A*_*t* + 1_.
6. Check the size of *A*_*t* + 1_. If *N*(*A*_*t* + 1_) > *N*_max_ then reduce *A*_*t* + 1_ by the truncation operator. If *N*(*A*_*t* + 1_) < *N*_max_ then fill *A*_*t* + 1_ with the best dominated individuals of *P*_*t*_ and *A*_*t*_, according to the fitness values.
7. If *t* ≥ *T* then create the A* set including the non-dominated individuals of *A*_*t* + 1_ and stop the procedure.
8. Perform a binary tournament selection with replacement between individuals of *A*_*t* + 1_ and fill the mating pool *K* with the winners until the size of *K* reaches *M*.
9. Apply mutation and crossover operators with the respective probabilities *p*_*m*_ and *p*_*c*_ to the individuals of the mating pool *K* and set *P*_*t* + 1_.
10. Increment *t*, i.e. *t* = *t* + 1 and go to step 4.

As the truncation operator we used the k-nearest neighbour method, which is a non-parametric classification method based on a similarity measure, e.g. distance functions [66]. According to [57], SPEA2 shows very good performance in comparison with other multi-objective optimization algorithms and can be easily adapted to various problems.

### The measures of distances between codes

To compare the SGC with the sets of codes optimized under the multi- and single-objectives, we proposed new measures (distances) describing differences in the objective function values of the genetic codes. In the case of the multi-objective optimization, every code was represented by a vector of eight values of the objective functions, whereas in the single-objective optimization, it was described by a single value of the fitness function. In the multi-objective case, we used two measures, *m*_*min*_ and *m*_*mean*_:

$$ {m}_{min}=\frac{db_{min}}{db_{min}+{dw}_{min}}\bullet 100\%, $$

where *db*_*min*_ is the minimum Euclidean distance in the eight-dimensional space of objective functions between the SGC and the codes minimizing amino acid replacement costs (the best codes), whereas *dw*_*min*_ is the minimum Euclidean distance in this space between the SGC and the codes maximizing the costs (the worst codes),

$$ {m}_{mean}=\frac{db_{mean}}{db_{mean}+{dw}_{mean}}\bullet 100\%, $$

where *db*_*mean*_ is the average Euclidean distance between the SGC and the (best) codes minimizing amino acid replacement costs, whereas *dw*_*mean*_ is the average Euclidean distance between the SGC and the (worst) codes maximizing the costs.

In the case of the single-objective optimization, we used the following measure:

$$ {m}_s=\frac{db}{ db w}\bullet 100\%, $$

where *db* is the distance between the SGC and the best code, whereas *dbw* is the distance between the best code and the worst code. This measure is similar to the percentage distance minimization proposed by M Di Giulio [37]. However, it does not determine the code optimality in relation to random codes but to the best and worst codes obtained by our algorithm.

All of these measures take values in the range from 0 to 100% and the values closer to zero indicate that the SGC is more similar to the best theoretical codes in terms of the robustness to the costs of amino acid replacements than to the worst codes. The inclusion of the worst codes in our approach enabled us to locate the SGC in the general space of possible genetic codes more accurately than it was previously done.

To assess the similarity (or lack thereof) between the SGC and the optimized codes, we used another measure of distance between the structures of two codes, *d*_*str*_, which is simply the number of codons having different amino acids assigned in these two codes:

$$ {d}_{str}\left(X,Y\right)={\sum}_{i=1}^{61}{d}_i^{X,Y}, $$

where *X* and *Y* are genetic codes and ${d}_i^{X,Y}=1$ if codon *i* encodes different amino acids in the codes *X* and *Y*. If the codon *i* has the same meaning, then ${d}_i^{X,Y}=0$. This measure is in fact a metric on a set of words with the same length, known as the Hamming distance; the smaller the number of the same assignments of amino acids to codons between two compared codes, the larger *d*_*str*_. Since we assumed that the stop translation codons do not change their meaning in the considered models of the genetic code, the maximum of *d*_*str*_ is 61.

### Simulation procedure

To find the codes that minimize or maximize the cost of amino acid replacements, we run MOEA-based simulations using a customized version of the SPEA2 algorithm [57]. We started with a population of *M* = 2800 randomly selected codes and we kept this number of codes in each consecutive generation. We also established the Pareto (archive) set to include up to *N*_max_ = 700 individuals. Each simulation was run up to *T* = 3000 steps (generations) and was repeated 20 times. In the simulations, we used the previously described fitness function, objective functions and genetic operators. The probabilities of mutation and crossover were set to 0.9 and 0.3, respectively, which means that in each step of the simulation we applied the mutation and crossover operators, one after the other, to, respectively, 90 and 30% of the individuals in the mating pool.

It seems natural to compare the outcome of multi-objective optimizations with the results of optimizations based on the same objectives but considered separately. Therefore, we also developed a single-objective customized evolutionary algorithm derived from the SPEA2 and we run it to find the optimized code for each of the eight objectives. Every simulation started also with a population of 2800 randomly chosen individuals. The probabilities of mutation and crossover were the same as in the case of the multi-objective optimization and the fitness function was analogous to the one used in the multi-objective case. The number of top optimized codes, selected for the archive set, was also set to 700. Each simulation was run up to 3000 steps and repeated 50 times.

In further parts of this paper, when we refer to the Pareto set obtained in any kind of multi-objective simulation, we mean a set of all the optimized codes combined from the respective repeated runs, i.e. 20 ∙ 700 = 14,000 codes. In the case of the single-objective optimization the final number of the optimized codes is 50 ∙ 700 = 35,000 codes.

## Results

### Optimality of the SGC in comparison to the optimized codes

The values of the proposed distance measures between the SGC and optimized codes, *m*_*min*_, *m*_*mean*_ and *m*_*s*_ can range from 0 to 100%. The values smaller than 50% indicate that the SGC shows a tendency to minimize rather than maximize the costs of amino acid replacements under a given criterion. The smaller the value, the more similar the optimality of the SGC to the best theoretical codes, i.e. minimizing the replacement costs. The values of the single-objective measure *m*_*s*_ are presented in Table 1.

**Table 1:** The values of the measure *m*_*s*_, which describe the relations between the SGC and the theoretical optimized codes obtained in the single-objective optimization algorithm under the BS and US models of the genetic code

Model	Objective	*m*_*S*_ [%]
BS	BIOV	16.16
	BLAM	57.47
	CEDJ	26.75
	LIFS	30.30
	MAXF	43.28
	MYIS	14.32
	NAKH	32.34
	TSAJ	31.63
US	BIOV	12.18
	BLAM	11.72
	CEDJ	13.37
	LIFS	15.48
	MAXF	18.02
	MYIS	13.29
	NAKH	15.82
	TSAJ	11.97

The same eight objectives were also used in the multi-objective optimization (Table 2). Values lower than 50% indicate that the SGC is closer to the theoretical code minimizing the costs in amino acid replacements rather than to the codes maximizing them. The objectives represent the following amino acid properties: BLAM - electric properties, BIOV - hydrophobicity, MAXF - alpha and turn propensities, TSAJ - physicochemical properties, NAKH - residue propensity, CEDJ - composition, LIFS - beta propensity and MIYS - intrinsic propensities

Only in the case of the BLAM index (representing electric properties) under the BS model, the standard genetic code is slightly closer to the codes that maximize this parameter than to those minimizing it (*m*_*s*_~ 57%). Interestingly, the measure *m*_*s*_ for the same index received the lowest value under the US model. In all other cases of the BS model, the values of *m*_*s*_ are lower than 50% and range from ~ 14% for the MYIS index to ~ 43% for the MAXF index. Under the US model, the range of *m*_*s*_ is narrower, from ~ 12% for the BIOV, BLAM and TSAJ indices to ~ 18% for the MYIS index. It should be noted that the values calculated under the US model are smaller because in a larger and less restricted search space it is possible to find the codes that maximize (and also minimize) the objective function to a greater extent than in the restricted space of the BS model. Thereby the denominator of *m*_*s*_ increases for the US model. These results suggest that the SGC is closer to the codes minimizing the cost than to the codes maximizing it regarding every single objective except for the already mentioned BLAM index under the BS model.

In the case of the multi-objective optimization tasks, we calculated the values of the *m*_*min*_ and *m*_*mean*_ measures (Table 2). Generally, under both the BS and US models, these values are similar and much lower than 50%, which indicates that the SGC is definitely closer to the codes minimizing the consequences of amino acid replacements than the codes maximizing them. The distances of the SGC to the best codes are slightly smaller under the less restrictive model of the genetic code. The average values of these measure are about two times larger than the minimum. It indicates that the SGC can minimize the cost of amino acid replacements quite similarly to some of the best theoretical codes but on average it is more distant from them because the Pareto front of the best equivalent codes is quite extensive.

**Table 2:** The values of the measures *m*_*min*_ and *m*_*mean*_, which describe the relations between the SGC and the Pareto sets of the theoretical optimized codes obtained from the multi-objective optimization algorithm under the BS and US models

Model	*m*_*min*_ [%]	*m*_*mean*_ [%]
BS	17.1	36.6
US	14.6	36.1

Values lower than 50% indicate that the SGC is closer to the set of codes minimizing the costs in amino acid replacements rather than the codes maximizing them

**Fig. 4:**
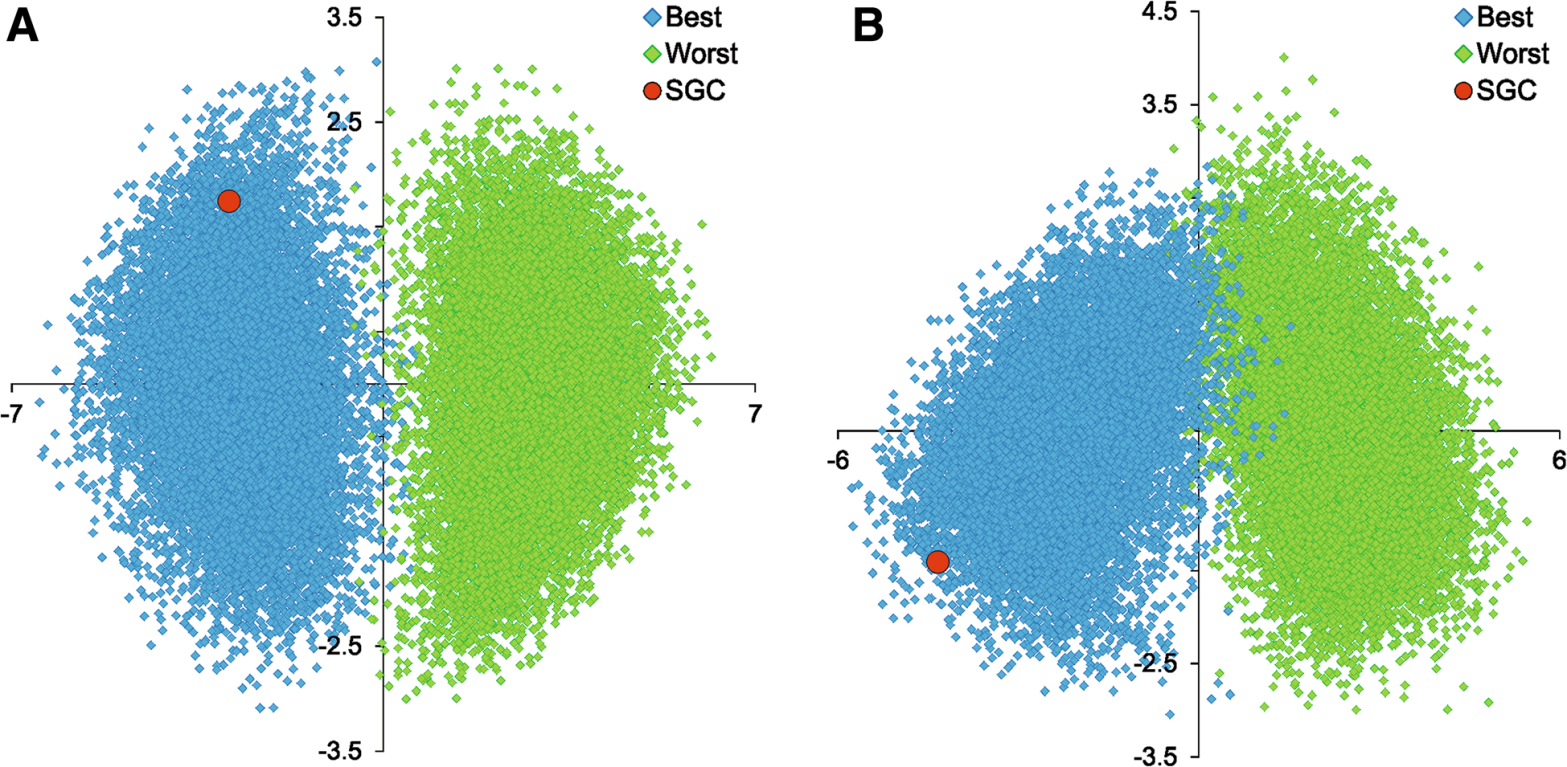
The plot of the discriminant function analysis based on the eight objective functions for the standard genetic code (SGC) as well as the codes minimizing (Best) and maximizing (Worst) the objective functions under the block structure (**a**) and unrestricted (**b**) models. The discriminant analysis was done in Statistica (StatSoft Inc. 2018, version 13, https://www.tibco.com/products/tibco-statistica)

To visualize the position of the SGC in the space of codes optimized under the multi-objective approach, we carried out discriminant analysis with canonical analysis [67] and presented the plot of two discriminant functions (Fig. 4). The theoretical codes are clearly separated into two sets, the best and the worst codes that were differently optimized, to minimize or to maximize the objective functions, respectively. The standard genetic code is placed definitely among the best codes but at the edge of their distribution. It suggests that only a small fraction of the best codes show the same properties as the SGC. According to the standardized function coefficients, the indices MAXF, TSAJ and NAKH have the greatest contribution to the first discriminant function (from 0.628 to 0.832) in the BS and US models. The second function is associated most with BIOV (0.985 and 1.239 in the BS and US models), MYIS (− 1.750 in the BS model) and NAKH (− 0.654 in the US model) indices. The factor structure coefficients indicate that the first discriminant function is weakly but positively correlated (from 0.153 to 0.350) with all indices in the two models, whereas the second function is most correlated with BIOV and MYIS, negatively (− 0.779 and − 0.798) in the BS model and positively (0.803 and 0.727) in the US model. These correlations and the position of the SGC in the plot imply that this code has a tendency to minimize these features.

Furthermore, we checked how many codes in the Pareto set obtained in the multi-objective optimization have lower values of the objective functions than the SGC, in other words, how many of them minimize the objective functions better than the SGC. The results are presented in Table 3.

**Table 3:** The number and percent of the codes in the Pareto set obtained in the multi-objective optimization under the BS and US models, which have the given number of the objective functions values smaller than the standard genetic code

Model	Number of objective functions	Number of codes	Percent of 14,000
BS	8	25	0.18
	7	117	0.84
	6	542	3.87
	5	1927	13.76
	4	4551	32.51
	3	4910	35.07
	2	1806	12.90
	1	117	0.84
US	8	1	0.01
	7	12	0.09
	5	302	2.16
	6	61	0.44
	4	1724	12.31
	3	5144	36.74
	2	5230	37.36
	1	1524	10.89

In the case of the BS model, out of the 14,000 codes only 25 were found better minimized than the standard genetic code for all 8 objectives. Most of the found codes (~ 94%) are able to minimize two to five objective functions better than the SGC. Under the US model, we found only one code which minimizes all 8 objectives better than the SGC. Most of the found codes (~ 97%) are characterized by a better minimization of one to four objective functions than the SGC. Obviously, the small number of the codes minimizing better all eight objectives may result from the difficulties in searching the huge space of potential codes with so many optimization criteria. However, in spite of these limitations, the results seem representative enough to justify the statement that it is possible to find the codes that are more robust to the costs of amino acid replacements than the SGC, when these cost are described by eight parameters. The number of better codes increases substantially when smaller number of parameters is considered. These results give another argument in favour of the opinion that the standard genetic code is clearly not our best option, but nevertheless it is still quite well optimized.

### Comparisons of objective functions for individual amino acid properties

**Fig. 5:**
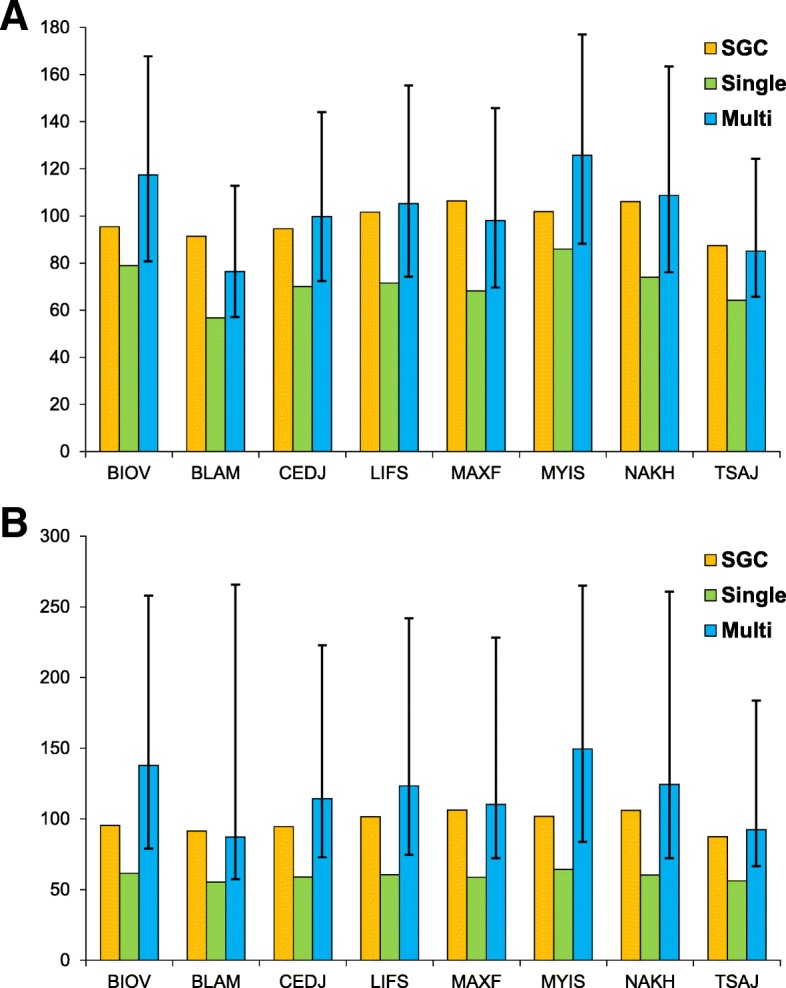
The values of the individual objective functions under the BS (**a**) and US (**b**) models for the SGC and the codes that minimize the functions in the single- and multi-objective optimizations. In the latter case, the blue bars show the average values of the function calculated for the codes in the Pareto set and the whiskers’ ends indicate the minimum and maximum values of this function. The objectives represent the following amino acid properties: BLAM - electric properties, BIOV - hydrophobicity, MAXF - alpha and turn propensities, TSAJ - physicochemical properties, NAKH - residue propensity, CEDJ - composition, LIFS - beta propensity and MIYS - intrinsic propensities

Since the single-objective algorithm is focused on optimization of only one objective function and the multi-objective algorithm optimizes many functions, we should expect that the genetic codes found in the former approach are better optimized for the individual function. To compare the results for each objective function corresponding to the respective amino acid index, we plotted their values for the SGC and for the best code found in the single-objective optimizations as well as the ranges of values of the respective objective functions for the best codes in the Pareto set obtained in the eight-objective optimization (Fig. 5).

We observed that under both the BS and US models the values of the objective functions are the smallest for the best code obtained in the single-objective optimizations, which could be expected (Fig. 5). However, the minimum values of the objective functions of the codes from the Pareto set obtained in the multi-objective optimization are only slightly greater. Under the BS model, the differences range from 0.26 in the case of the BLAM index to 2.73 in the case of the LIFS index; under the US model they range from 2.06 in the case of the BLAM index to 19.5 in the case of the MYIS index. These differences are relatively small because the values of the objective functions for the best codes found by the multi-objective optimization algorithm are only 1.005 to 1.303 times greater than the respective values obtained in the single-objective optimization. It proves that using multi-objective optimization methods we can find codes which are almost as optimal as those obtained by the single-objective optimization algorithms regarding a given objective. However, the codes found by the multi-objective methods have the additional advantage of being optimized also with regard to other criteria. For example, under the US model one of the codes in the Pareto set obtained in the multi-objective optimization has the value of the objective function BLAM equal to 59.922 whereas the value for the best code optimized only for the BALM index is merely slightly smaller: 55.439. However, the values of the other objective functions for the code found by the multi-objective optimization algorithm indicate that it is better optimized regarding five out of eight objectives: BIOV, CEDJ, LIFS, MAXF and MYIS (Table 4).

**Table 4:** The values of the objective functions for the best code optimized to minimize only the BLAM objective costs (single-objective) and for one of the codes in the Pareto set obtained in multi-objective optimization (multi-objective). Both codes were optimized under the US model

Objective	BIOV	BLAM	CEDJ	LIFS	MAXF	MYIS	NAKH	TSAJ
single	108.53	55.439	110.73	88.357	131.26	112.56	90.517	84.602
multi	100.702	59.922	97.336	77.79	96.107	100.825	102.578	97.236

The values of the objective functions for the SGC are only 1.2 to 1.8 times greater than the respective values for the codes optimized to minimize one objective (Fig. 5) and 1.2 to 1.6 times greater than the respective values for the best codes optimized to minimize all the eight objectives simultaneously. However, in many cases, the values of the objective functions for the SGC are smaller than the average values for the codes of the Pareto set obtained in the multi-objective optimization; they are greater only for the BLAM index under both models of codes as well as for the MAXF and TSAJ indices under the BS model. Moreover, the function values of the SGC never exceed the maximum values obtained for codes optimized in the multi-objective approach. These observations suggest that the standard genetic code is quite well optimized regarding the eight objectives considered in this study, although it is not perfect.

### Comparison of the genetic codes structures

To check how much the structure of the optimized codes obtained in the multi-objective optimization is different from the SGC, we applied the measure *d*_*str*_. It shows the number of codons which have different amino acids assignments in two compared codes. The maximum possible value of *d*_*str*_ is 61 because we fixed the meaning of the stop translation codons as it is in the SGC. For both the BS and US models, we considered two groups of codes: (i) the ones (called Group 8) characterized by the values of the eight objective functions smaller than the SGC and (ii) all the codes from the Pareto set of optimal solutions. The median values of *d*_*str*_ are equal to 58 or 59 in both groups of codes and under both models, which means that the optimized codes have usually only few assignments of codons to amino acids the same as the SGC.

**Fig. 6:**
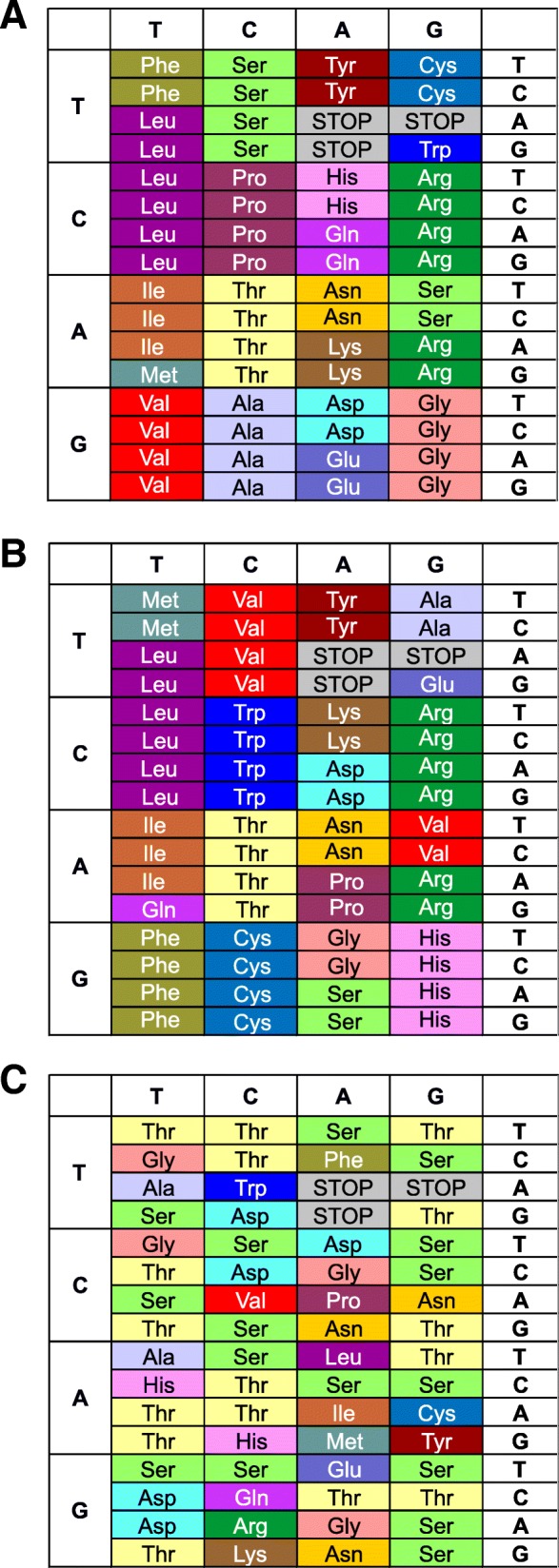
The standard genetic code (**a**), the code which was found under the BS model and is the most similar structurally to the SGC (**b**), the only code found under the US model, which has the values of all eight objective functions smaller than the SGC (**c**)

The code most similar structurally to the SGC is among the whole Pareto set optimized under the BS model. However, it still has 38 different assignments (Table 5). This code retained the same codon blocks of leucine, isoleucine, threonine, tyrosine, asparagine and arginine as are in the SGC (Fig. 6b). Lysine and aspartic acid kept the same number of codons and stayed in the same column as in the SGC. However, the other 12 amino acids changed their codon numbers and positions in the code table. The greatest difference refers to serine, whose number of codons was reduced from six to two, and tryptophan, for which the number of codons increased from one to four.

**Table 5:** The minimum, median and maximum values of the measure *d*_*str*_ for all the codes in the Pareto sets of optimal solutions and for the codes with all eight values of the objective functions smaller than in the case of the SGC (Group 8), under the BS and US models

Model	Subgroup	Total number of codes	Structure distance *d*_*str*_		
			min	median	max
BS	Group 8	25	51	59	61
	Pareto set	14,000	38	59	61
US	Group 8	1	58	58	58
	Pareto set	14,000	51	59	61

The only code found under the US model, which has the values of all 8 objective functions smaller than the SGC, is much different from the standard genetic code (Fig. 6c). Only the codons ACC and ACA for threonine and AGC for serine have the same meaning as in the SGC. Serine and threonine are also assigned to considerably more codons, i.e. 16, than the others. Aspartic acid has five codons assigned, asparagine three, glycine four, alanine and histidine two, whereas the remaining amino acids are assigned to single codons. The codon block structure typical of the SGC is not well represented in this code. Many codons encoding the same amino acid are not adjacent in the code table. The largest block of degenerated codons associated with the third codon position consists of three codons for serine. Besides that, there are eight two-fold degenerated codons for serine, threonine and aspartic acid. The other cases of degeneration are related to the first and the second codon positions, e.g. there are three blocks with three codons each which can encode serine and threonine regardless of the nucleotide in the first codon position. Two such three-codon blocks degenerated in terms of the second codon position encode also serine and threonine.

The predominance of serine and threonine in the best code may follow from the fact that the values of their properties are very close to the average values for all amino acids. Then, the cost of replacements between amino acids is minimized. The same was observed in the case of polarity, when the best code was dominated by alanine, serine and glycine [51]. To verify it in the multi-objective case, we calculated the absolute differences between the average value of each amino acid index considered in our optimization, and the respective values for all 20 amino acids (Table 6).

**Table 6:** The absolute differences between the average values of a given index and the individual values of this index for each amino acid

Amino acid	Amino acid index								Average	Number of codons
	BLAM	BIOV	MAXF	TSAJ	NAKH	CEDJ	LIFS	MYIS		
Ala	0.516	0.043	0.243	0.239	0.262	0.337	0.062	0.067	0.221	2
Arg	0.318	0.498	0.093	0.207	0.284	0.012	0.057	0.400	0.234	1
Asn	0.078	0.519	0.230	0.093	0.162	0.116	0.239	0.582	0.252	3
Asp	0.047	0.540	0.062	0.128	0.244	0.058	0.305	0.764	0.269	5
Cys	0.047	0.762	0.050	0.181	0.147	0.360	0.070	0.964	0.323	1
Gln	0.349	0.513	0.117	0.015	0.232	0.070	0.046	0.612	0.244	1
Glu	0.068	0.688	0.387	0.020	0.244	0.244	0.233	0.885	0.346	1
Gly	0.484	0.196	0.338	0.352	0.070	0.244	0.233	0.461	0.297	4
His	0.109	0.137	0.026	0.062	0.272	0.337	0.057	0.067	0.133	2
Ile	0.391	0.672	0.010	0.087	0.525	0.023	0.430	0.842	0.372	1
Leu	0.474	0.640	0.201	0.087	0.700	0.419	0.148	0.933	0.450	1
Lys	0.276	0.873	0.147	0.096	0.266	0.198	0.184	1.188	0.403	1
Met	0.411	0.529	0.303	0.099	0.064	0.302	0.087	0.721	0.315	1
Phe	0.130	0.873	0.099	0.209	0.360	0.128	0.120	0.964	0.360	1
Pro	3.089	0.233	0.320	0.096	0.183	0.035	0.349	0.764	0.634	1
Ser	0.068	0.498	0.194	0.217	0.035	0.186	0.117	0.552	0.233	16
Thr	0.078	0.328	0.146	0.105	0.019	0.035	0.048	0.279	0.130	16
Trp	0.120	0.640	0.008	0.367	0.168	0.442	0.280	0.812	0.355	1
Tyr	0.266	0.153	0.200	0.226	0.112	0.221	0.275	0.691	0.268	1
Val	0.172	0.524	0.026	0.023	0.445	0.209	0.424	0.691	0.314	1

Since the indices were in different scales, they were at first normalized by their maximum values. The column next to the last contains the average calculated from the values in a row. The last column contains the number of codons for the given amino acid in the best code that was found under the US model and has the values of all eight objective functions smaller than the SGC

In the cases of the BLAM and NAKH indices, serine and threonine have their values of these indices close to the average values. This tendency is also present for threonine in the LIFS and CEDJ indices. In consequence, threonine has on average the smallest deviation from average values of all indices (Table 6). Generally, there is a significant negative Spearman correlation between the number of codons assigned to a given amino acid and the average of the absolute differences from the eight amino acid indices: − 0.659 (*p*-value: 0.0016). Therefore, a code having amino acids with ‘average’ properties assigned to large number of codons minimizes the costs of replacements with other amino acids, especially those with large values of the given indices. This property is not present in the SGC because this coefficient is − 0.163 and is not statistically significant (p-value, 0.49).

**Fig. 7:**
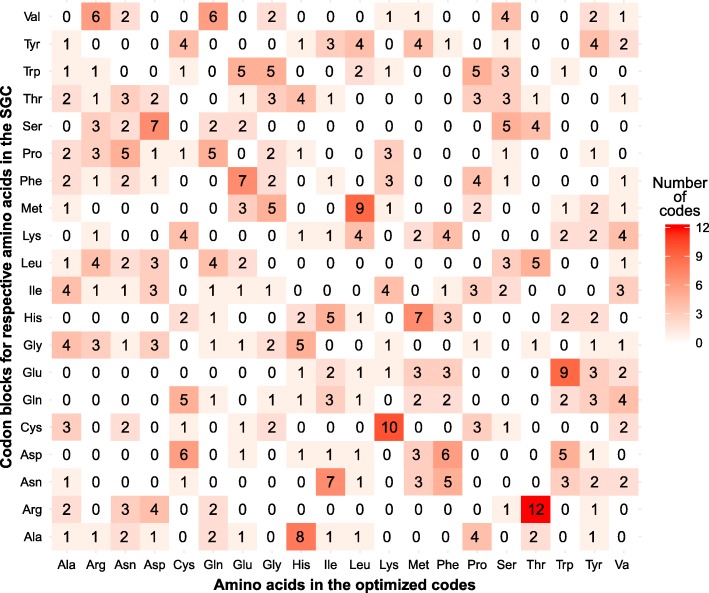
A heatmap presenting the numbers of codes which have a given amino acid (horizontal axis) assigned to the particular codon block (vertical axis). The data were obtained for 25 codes from the Pareto set under the BS model, which have the values of the eight objective functions better minimized than the standard genetic code

Finally, to compare the 25 codes that were found under the BS model and that minimize all eight objective functions better than the SGC, we calculated how many of these codes have amino acids assigned to particular codon blocks as in the SGC. The results are presented in Fig. 7. The same assignments of amino acids to codons as in the SGC occur very rarely in the optimized codes. The encoding of serine and tyrosine was not changed only in five and four optimized codes, respectively. We can also notice that the most frequent assignment in the optimized codes was that of threonine to the codon block linked with arginine in the standard code. It occurred 12 times. Threonine was also five times assigned to the codon block of leucine and four times to the serine codon block. Interestingly, the codon blocks of arginine, leucine and serine are composed of six codons in the SGC, which is the largest possible number of codons in a block in the natural code (Fig. 6a). Such assignment of threonine to the large number of codons minimizes the costs of its replacements with other amino acids and vice versa because this amino acid shows the smallest deviation from the average indices values of all amino acids (Table 6). Threonine is also widely distributed in the best code found under the US model (Fig. 6c). Histidine, another amino acid with the small deviation in the indices (Table 6) is encoded by two codons in the SGC (Fig. 6a) but in the best codes of the BS model, it was assigned 17 times to four-codon blocks, which also increased its representation in the optimized codes (Fig. 7).

Besides the assignment of threonine to arginine codons, the next frequent assignments in the best codes of the BS model are: lysine to cysteine codons (in 10 codes), tryptophan to glutamic acid codons (in nine codes) and leucine to methionine codons (in nine codes) (Fig. 7). The last case is a reduction of the codon number from six to only one and may be associated with the fact that this amino acid is characterized by highly hydrophobic properties and has the values of most amino acid indices strongly deviated from the average (Table 6). Leucine was additionally assigned to one tryptophan codon in two of the optimized codes and 13 times to two-codons blocks of other amino acids. In general, the other amino acids with values of most amino acid indices different from the average value were much more frequently assigned to one- or two-codons blocks than to blocks consisting of more than two codons. Such assignments of these amino acids help in minimizing the costs of replacements with other amino acids and vice versa.

## Discussion

The results presented in this work uncovered interesting aspects of the standard genetic code optimality using new approaches. In contrast to the previous methods, which used mainly randomly generated codes as a reference to the SGC [24–27, 44], we compared this code with not only the best, but also the worst alternatives maximizing the probability of harmful changes in proteins. To find the optimal codes, we applied a specific version of an evolutionary algorithm, which seems to be a better approach in the study of the genetic code optimization than the classic comparison of the SGC with randomly generated theoretical alternatives due to the large number of possible codes and the extremely vast search space [37, 45–52]. The random codes represent only a very tiny fraction of all possibilities and are not necessarily representative of the whole space of the theoretical codes. Moreover, depending on the randomization method, the generated codes can be characterized by relatively uniform or biased amino acid assignments to codons. In comparison with the randomly generated codes, the SGC turned out to be quite robust to mutations and mistranslations [24, 26, 27, 30, 68–70]. However, when evolutionary algorithms were applied to find the most robust codes, the SGC turned out much less optimized to minimize the mutational and translational errors [38, 47, 49, 51]. Our findings confirm the second conclusion.

So far, the code optimality was usually studied in terms of only few single amino acid properties, mainly polar requirements [24, 37, 41, 47, 50, 55, 56]. However, it is obvious that if such optimization in fact occurred, many features of amino acids must have influenced the SGC evolution. Therefore, we applied a more general approach which took into account more than one property of amino acids. To avoid an arbitrary choice of the amino acids features, we considered the amino acid indices representing eight clusters of more than 500 various amino acid properties. Therefore, we can assume that the selected parameters are representative of the most relevant amino acid properties. As a result, we were able to investigate the SGC optimality under relatively general conditions and without arbitrary constraints.

The presented outcomes demonstrate that it is hard to interpret the properties of the SGC unambiguously. On the one hand, it could be significantly improved in all considered parameters. There is a substantial fraction of codes that minimize the amino acid replacement effects better than the SGC according to several amino acid properties simultaneously. Moreover, the structures of the best genetic codes differ substantially from the SGC structure, which indicates that the full optimization of the consequences of amino acid replacements can be achieved by a completely different assignments of amino acids to codons. The optimized structures are dominated by amino acids with average physicochemical properties. Therefore, we can state that the standard genetic code is not fully optimized in this respect. It is possible that the addition of amino acids with extreme properties to the genetic code during its expansion was more favourable than the potential benefits resulting from the minimization of mutational and translational errors in proteins. The amino acids with disparate features could provide new properties and functionality of translated peptides and proteins. On the other hand, using new types of measures placing the SGC in the global space of the theoretical codes and taking into consideration not only the best but also the worst possible genetic codes, we observe that the SGC has nevertheless a strong tendency to minimize the costs of potential amino acid replacements under different and often mutually exclusive criteria. The SGC appears to minimize the costs related to hydration potential, refractivity, optical activity, flexibility and hydrophobicity but it is very poorly optimized according to electric properties.

Our findings correspond to other results suggesting that the SGC is only partially optimized and is not located even in a deep local minimum [38, 51, 71]. Various models focusing on the genetic code expansion and occupation of codons by amino acids were proposed. However, a genetic code minimizing mutational and translational errors did not have to be directly selected under these scenarios. According to one of them, the SGC could have been derived from a primeval code consisting of RNY (R-purine, Y-pyrimidine) codons [72]. Next, additional codons could have been generated in the second (NYR) and the third (YRN) reading frames as well as by transversions in the first (YNY) and third (RNR) codon positions [73, 74]. Other models assume that the reduction of codon ambiguity resulted from successive binary choices based on distinct properties of two classes of aminoacyl-tRNA synthetases, which aminoacylated tRNAs differently [75, 76]. A specific complementarity in tRNAs and these synthetases could also have contributed to the SGC evolution [75]. It was also postulated that the SGC started from GNN codons and rapidly developed into a four-column code [77]. In this model, amino acids were assigned to codons to minimize the disruption of already existing proteins. In agreement with the coevolution hypothesis, the early code also consisted of GNN codons but amino acids were assigned to codons according to the development of biosynthetic pathways [19]. In the 2–1-3 model, the coding specificity was successively increased in individual codon positions in the order: the second, the first and the third codon position [78, 79].

Assuming the correctness of these models, it is not inconceivable that the minimization property of the SGC could have evolved as a by-product of evolution without a direct selection on this feature and it could have been driven by other factors, e.g. specific additions of amino acids to the code to minimize damages in already encoded proteins [77], the diversification of the repertoire of amino acids in proteins [7, 77, 80, 81], biosynthetic pathways [14–19] or the duplication of genes for tRNAs and aminoacyl-tRNA synthetases [7, 79, 82–86] as well as their coevolution [87]. According to these concepts, the physicochemical properties of amino acid played only a subsidiary role in the SGC evolution [20, 21, 88], whereas the harmful effects of mutations were minimized mainly by the direct optimization of the mutational pressure [29, 89–91]. When the SGC reached a given evolutionary stage, it was fixed [40], which could have prevented its full optimization and reassignments of already introduced amino acid to the code because any substantial reassignments would be lethal [3].

Hence the question about the main forces responsible for the present structure of the standard genetic code still remains open. However, our results are a good motivation for the future studies on this problem. It seems clear that the SGC did not evolve to optimize only one selected property, but there must have been several different factors involved. Therefore, the multi-objective optimization is a justifiable, if not necessary, approach. Future studies should take into account other objectives related to the genetic code adaptability.

Our findings can be very useful to researchers modifying the genetic code of the living organisms and designing artificial ones [92–96]. The knowledge of which assignments of codons to amino acid are beneficial to the organism and which could be changed to improve the desired characteristic is vital in this line of research. Such modifications can be used to produce peptides or proteins including unnatural amino acids and showing novel properties.

## Abbreviations

BS: The block structure model of the genetic code
MOEAs: The multi-objective evolutionary algorithms
SGC: Standard genetic code
SPEA2: Strength Pareto Evolutionary Algorithm
US: The unrestricted structure model of the genetic code

## References

[1] Khorana HG, Buchi H, Ghosh H, Gupta N, Jacob TM, Kossel H, Morgan R, Narang SA, Ohtsuka E, Wells RD (1966). Polynucleotide synthesis and the genetic code. Cold Spring Harb Symp Quant Biol.

[2] Nirenberg M, Caskey T, Marshall R, Brimacombe R, Kellogg D, Doctor B, Hatfield D, Levin J, Rottman F, Pestka S (1966). The RNA code and protein synthesis. Cold Spring Harb Symp Quant Biol.

[3] Crick FH (1968). The origin of the genetic code. J Mol Biol.

[4] Di Giulio M (2005). The origin of the genetic code: theories and their relationships, a review. Biosystems.

[5] Knight RD, Freeland SJ, Landweber LF (1999). Selection, history and chemistry: the three faces of the genetic code. Trends Biochem Sci.

[6] Koonin EV, Novozhilov AS (2009). Origin and evolution of the genetic code: the universal enigma. IUBMB Life.

[7] Koonin EV, Novozhilov AS (2017). Origin and evolution of the universal genetic code. Annu Rev Genet.

[8] Dunnill P (1966). Triplet nucleotide-amino-acid pairing - a stereochemical basis for division between protein and non-protein amino-acids. Nature.

[9] Pelc SR, Welton MGE (1966). Stereochemical relationship between coding triplets and amino-acids. Nature.

[10] Woese CR (1968). Fundamental nature of genetic code - prebiotic interactions between polynucleotides and polyamino acids or their derivatives. Proc Natl Acad Sci U S A.

[11] Yarus M, Caporaso JG, Knight R (2005). Origins of the genetic code: the escaped triplet theory. Annu Rev Biochem.

[12] Yarus M, Widmann JJ, Knight R (2009). RNA-amino acid binding: a stereochemical era for the genetic code. J Mol Evol.

[13] Woese CR, Dugre DH, Saxinger WC, Dugre SA (1966). Molecular basis for genetic code. Proc Natl Acad Sci U S A.

[14] Wong JT (1975). A co-evolution theory of the genetic code. Proc Natl Acad Sci U S A.

[15] Wong JT, Ng SK, Mat WK, Hu T, Xue H (2016). Coevolution theory of the genetic code at age forty: pathway to translation and synthetic life. Life (Basel).

[16] Di Giulio M (1997). On the origin of the genetic code. J Theor Biol.

[17] Di Giulio M (1999). The coevolution theory of the origin of the genetic code. J Mol Evol.

[18] Di Giulio M (2004). The coevolution theory of the origin of the genetic code. Phys Life Rev.

[19] Di Giulio M (2008). An extension of the coevolution theory of the origin of the genetic code. Biol Direct.

[20] Di Giulio M (2016). The lack of foundation in the mechanism on which are based the physico-chemical theories for the origin of the genetic code is counterposed to the credible and natural mechanism suggested by the coevolution theory. J Theor Biol.

[21] Di Giulio M (2017). Some pungent arguments against the physico-chemical theories of the origin of the genetic code and corroborating the coevolution theory. J Theor Biol.

[22] Sonneborn TM, Bryson V, Vogel HJ (1965). Degeneracy of the genetic code: extent, nature, and genetic implications. Evolving genes and proteins.

[23] Woese CR (1965). On the evolution of the genetic code. Proc Natl Acad Sci U S A.

[24] Haig D, Hurst LD (1991). A quantitative measure of error minimization in the genetic code. J Mol Evol.

[25] Freeland SJ, Hurst LD (1998). Load minimization of the genetic code: history does not explain the pattern. Proc R Soc B Biol Sci.

[26] Freeland SJ, Hurst LD (1998). The genetic code is one in a million. J Mol Evol.

[27] Gilis D, Massar S, Cerf NJ, Rooman M (2001). Optimality of the genetic code with respect to protein stability and amino-acid frequencies. Genome Biol.

[28] Freeland SJ, Wu T, Keulmann N (2003). The case for an error minimizing standard genetic code. Orig Life Evol Biosph.

[29] Mackiewicz P, Biecek P, Mackiewicz D, Kiraga J, Baczkowski K, Sobczynski M, Cebrat S (2008). Optimisation of asymmetric mutational pressure and selection pressure around the universal genetic code. Comput Sci - ICCS.

[30] Freeland SJ, Knight RD, Landweber LF, Hurst LD (2000). Early fixation of an optimal genetic code. Mol Biol Evol.

[31] Epstein CJ (1966). Role of the amino-acid “code” and of selection for conformation in the evolution of proteins. Nature.

[32] Goodarzi H, Najafabadi HS, Nejad HA, Torabi N (2005). The impact of including tRNA content on the optimality of the genetic code. Bull Math Biol.

[33] Goldberg AL, Wittes RE (1966). Genetic code: aspects of organization. Science.

[34] Ardell DH (1998). On error minimization in a sequential origin of the standard genetic code. J Mol Evol.

[35] Ardell DH, Sella G (2001). On the evolution of redundancy in genetic codes. J Mol Evol.

[36] Di Giulio M, Medugno M (1999). Physicochemical optimization in the genetic code origin as the number of codified amino acids increases. J Mol Evol.

[37] Di Giulio M (1989). The extension reached by the minimization of the polarity distances during the evolution of the genetic code. J Mol Evol.

[38] Novozhilov AS, Wolf YI, Koonin EV (2007). Evolution of the genetic code: partial optimization of a random code for robustness to translation error in a rugged fitness landscape. Biol Direct.

[39] García JA, Alvarez S, Flores A, Govezensky T, Bobadilla JR, José MV (2004). Statistical analysis of the distribution of amino acids in Borrelia burgdorferi genome under different genetic codes. Phys A Stat Mech Appl.

[40] Zamudio GS, Jose MV (2018). Phenotypic graphs and evolution unfold the standard genetic code as the optimal. Origins Life Evol Biospheres.

[41] Błażej P, Wnętrzak M, Mackiewicz D, Mackiewicz P (2018). Optimization of the standard genetic code according to three codon positions using an evolutionary algorithm. PLoS One.

[42] Schönauer S, Clote P, Frishman D, Mewes HW (1997). How optimal is the genetic code?. Computer Science and Biology Proceedings of the German Conference on Bioinformatics (GCB’97) Sep 21–24.

[43] Zamudio GS, Jose MV (2017). On the Uniqueness of the Standard Genetic Code. Life (Basel).

[44] Freeland SJ, Knight RD, Landweber LF (2000). Measuring adaptation within the genetic code. Trends Biochem Sci.

[45] Di Giulio M. The origin of the genetic code. Trends Biochem Sci. 2000;25(2):44–4. 10.1016/s0968-0004(99)01522-4 10664578

[46] Di Giulio M, Capobianco MR, Medugno M. On the optimization of the physicochemical distances between amino-acids in the evolution of the genetic-code. J Theor Biol. 1994;168(1):43–51. 10.1006/jtbi.1994.1086 8022190

[47] Judson OP, Haydon D (1999). The genetic code: what is it good for? An analysis of the effects of selection pressures on genetic codes. J Mol Evol.

[48] Santos J, Monteagudo A (2010). Study of the genetic code adaptability by means of a genetic algorithm. J Theor Biol.

[49] Santos J, Monteagudo A (2011). Simulated evolution applied to study the genetic code optimality using a model of codon reassignments. BMC Bioinf.

[50] de Oliveira LL, de Oliveira PS, Tinos R (2015). A multiobjective approach to the genetic code adaptability problem. BMC Bioinf.

[51] Błażej P, Wnętrzak M, Mackiewicz P (2016). The role of crossover operator in evolutionary-based approach to the problem of genetic code optimization. Biosystems.

[52] de Oliveira LL, Freitas AA, Tinós R (2018). Multi-objective genetic algorithms in the study of the genetic code’s adaptability. Inf Sci.

[53] Di Giulio M (2001). The origin of the genetic code cannot be studied using measurements based on the PAM matrix because this matrix reflects the code itself, making any such analyses tautologous. J Theor Biol.

[54] Rudnicki WR, Mroczek T, Cudek P (2014). Amino acid properties conserved in molecular evolution. PLoS One.

[55] Di Giulio M (1989). Some aspects of the organization and evolution of the genetic code. J Mol Evol.

[56] Xia X, Li W (1998). What amino acid properties affect protein evolution?. J Mol Evol.

[57] Zitzler E, Laumanns M, Thiele L, Giannakoglou KC, Tsahalis DT, Periaux J, Papailiou KD, Barcelona FT (2002). SPEA2: improving the strength Pareto evolutionary algorithm for multiobjective optimization. Evolutionary methods for design, optimisation and control with application to industrial problems proceedings o/the EUROGEN2001 conference, Athens, Greece, September 19–21,2001.

[58] Kawashima S, Pokarowski P, Pokarowska M, Kolinski A, Katayama T, Kanehisa M (2008). AAindex: amino acid index database, progress report 2008. Nucleic Acids Res.

[59] Saha I, Maulik U, Bandyopadhyay S, Plewczynski D (2012). Fuzzy clustering of physicochemical and biochemical properties of amino acids. Amino Acids.

[60] Sivanandam SN, Deepa SN (2008). Introduction to genetic algorithms.

[61] Syswerda G, Davis L (1991). Schedule optimization using genetic algorithms. Handbook of genetic algorithms.

[62] Coello Coello C, Lamont GB, van Veldhuizen D (2007). Evolutionary algorithms for solving multi-objective problems.

[63] Corus D, Dang DC, Eremeev AV, Lehre PK (2018). Level-based analysis of genetic algorithms and other search processes. IEEE Trans Evol Comput.

[64] Oliveto PS, Witt C (2014). On the runtime analysis of the simple genetic algorithm. Theor Comput Sci.

[65] Oliveto PS, Witt C (2015). Improved time complexity analysis of the simple genetic algorithm. Theor Comput Sci.

[66] Cunningham P, Delany S (2007). K-nearest neighbour classifiers. Multi-Classifier Systems.

[67] McLachlan G (1992). Discriminant analysis and statistical pattern recognition.

[68] Buhrman H, van der Gulik PTS, Kelk SM, Koolen WM, Stougie L. Some Mathematical Refinements Concerning Error Minimization in the Genetic Code. IEEE-ACM Trans Comput Biol Bioinform. 2011;8(5):1358–72. 10.1109/TCBB.2011.40 21358008

[69] Goodarzi H, Nejad HA, Torabi N (2004). On the optimality of the genetic code, with the consideration of termination codons. Biosystems.

[70] Sella G, Ardell DH (2006). The coevolution of genes and genetic codes: Crick’s frozen accident revisited. J Mol Evol.

[71] Santos J, Monteagudo A (2017). Inclusion of the fitness sharing technique in an evolutionary algorithm to analyze the fitness landscape of the genetic code adaptability. BMC Bioinf.

[72] Eigen M, Schuster P (1978). Hypercycle - principle of natural self-organization .B. Abstract Hypercycle. Naturwissenschaften.

[73] Jose MV, Govezensky T, Garcia JA, Bobadilla JR (2009). On the evolution of the standard genetic code: vestiges of critical scale invariance from the RNA world in current prokaryote genomes. PLoS One.

[74] Jose MV, Zamudio GS, Morgado ER (2017). A unified model of the standard genetic code. R Soc Open Sci.

[75] Rodin SN, Rodin AS (2008). On the origin of the genetic code: signatures of its primordial complementarity in tRNAs and aminoacyl-tRNA synthetases. Heredity (Edinb).

[76] Delarue M (2007). An asymmetric underlying rule in the assignment of codons: possible clue to a quick early evolution of the genetic code via successive binary choices. RNA.

[77] Higgs PG (2009). A four-column theory for the origin of the genetic code: tracing the evolutionary pathways that gave rise to an optimized code. Biol Direct.

[78] Massey SE (2006). A sequential “2-1-3” model of genetic code evolution that explains codon constraints. J Mol Evol.

[79] Massey SE (2008). A neutral origin for error minimization in the genetic code. J Mol Evol.

[80] Sengupta S, Higgs PG (2015). Pathways of genetic code evolution in ancient and modern organisms. J Mol Evol.

[81] Weberndorfer G, Hofacker IL, Stadler PF (2003). On the evolution of primitive genetic codes. Orig Life Evol Biosph.

[82] Massey SE (2015). Genetic code evolution reveals the neutral emergence of mutational robustness, and information as an evolutionary constraint. Life (Basel).

[83] Massey SE (2016). The neutral emergence of error minimized genetic codes superior to the standard genetic code. J Theor Biol.

[84] Koonin EV (2017). Frozen accident pushing 50: stereochemistry, expansion, and chance in the evolution of the genetic code. Life (Basel).

[85] Cavalcanti AR, Leite ES, Neto BB, Ferreira R (2004). On the classes of aminoacyl-tRNA synthetases, amino acids and the genetic code. Orig Life Evol Biosph.

[86] Cavalcanti AR, Neto BD, Ferreira R (2000). On the classes of aminoacyl-tRNA synthetases and the error minimization in the genetic code. J Theor Biol.

[87] de Farias ST, do Rego TG, Jose MV (2014). Evolution of transfer RNA and the origin of the translation system. Front Genet.

[88] Di Giulio M (2013). The origin of the genetic code: matter of metabolism or physicochemical determinism?. J Mol Evol.

[89] Dudkiewicz A, Mackiewicz P, Nowicka A, Kowalezuk M, Mackiewicz D, Polak N, Smolarczyk K, Banaszak J, Dudek MR, Cebrat S (2005). Correspondence between mutation and selection pressure and the genetic code degeneracy in the gene evolution. Futur Gener Comput Syst.

[90] Błażej P, Miasojedow B, Grabińska M, Mackiewicz P (2015). Optimization of mutation pressure in relation to properties of protein-coding sequences in bacterial genomes. PLoS One.

[91] Błażej P, Mackiewicz D, Grabińska M, Wnętrzak M, Mackiewicz P (2017). Optimization of amino acid replacement costs by mutational pressure in bacterial genomes. Sci Rep.

[92] Zhang Y, Ptacin JL, Fischer EC, Aerni HR, Caffaro CE, San Jose K, Feldman AW, Turner CR, Romesberg FE (2017). A semi-synthetic organism that stores and retrieves increased genetic information. Nature.

[93] Lajoie MJ, Rovner AJ, Goodman DB, Aerni HR, Haimovich AD, Kuznetsov G, Mercer JA, Wang HH, Carr PA, Mosberg JA (2013). Genomically recoded organisms expand biological functions. Science.

[94] Lee BS, Kim S, Ko BJ, Yoo TH (2017). An efficient system for incorporation of unnatural amino acids in response to the four-base codon AGGA in Escherichia coli. Biochim Biophys Acta Gen Subj.

[95] Lin X, Yu AC, Chan TF (2017). Efforts and challenges in engineering the genetic code. Life (Basel).

[96] Chin JW (2014). Expanding and reprogramming the genetic code of cells and animals. Annu Rev Biochem.

